# Creation of Coronary Conduit and Neo-Ostium in Previously Ligated Left Coronary Artery With Aortic Homograft

**DOI:** 10.1016/j.jaccas.2025.106505

**Published:** 2025-12-17

**Authors:** Jarrod D. Frizzell, Nadia El Hangouche

**Affiliations:** The Christ Hospital, Cincinnati, Ohio, USA

**Keywords:** computed tomography, intravascular ultrasound, percutaneous coronary intervention

## Abstract

**Case Summary:**

A 69-year-old man with a complex cardiac history including prosthetic aortic valve endocarditis with abscess involving the left coronary artery requiring placement of an aortic root homograft with coronary bypass grafting and ligation of the left coronary presented with severe angina following graft failure with severe native vessel disease. Retrograde chronic total occlusion percutaneous coronary intervention techniques, including electrocautery-assisted reentry, were used to connect the disrupted artery with the aortic root.

**Take-Home Message:**

Chronic total occlusion percutaneous coronary intervention techniques may facilitate reconnection of surgically disrupted coronary arteries with the aortic root.

## Graphical Abstract

**Figure:**
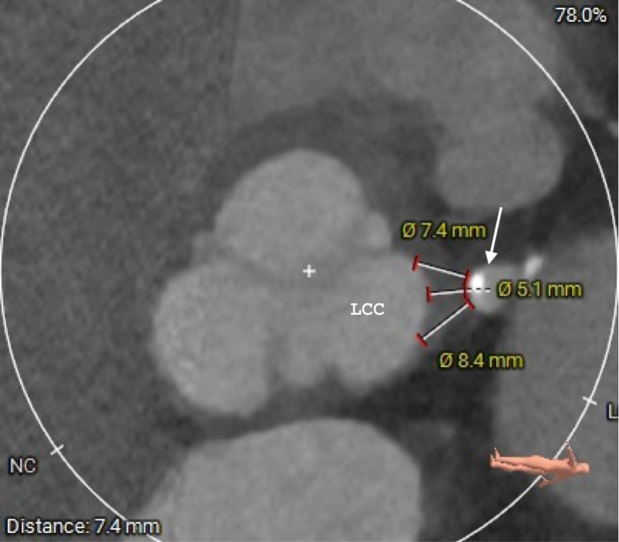

Although chronic total occlusion (CTO) percutaneous coronary intervention (PCI) has made significant gains over the years, to date, none have described extension of these techniques to facilitate percutaneous revascularization of surgically disrupted coronary arteries. We describe a novel application of CTO PCI techniques in a patient with refractory angina and inhospitable anatomy.

**Take-Home Message**

• Use of chronic total occlusion percutaneous coronary intervention techniques, specifically retrograde electrocautery-assisted reentry, may be used to create aortocoronary connections in previously ligated coronary arteries.

A 69-year-old man with complex cardiovascular history presented with new-onset rapidly progressive angina, now class IV. He had surgical aortic valve replacement 15 years prior for severely stenotic biscuspid aortic valve and a redo surgical aortic valve replacement for valvular degeneration 5 years prior to this presentation. A year following his last operation, he had prosthetic aortic valve endocarditis with annular abscess, complicated by septic emboli that ultimately resulted in stroke and left below-the-knee amputation. Emergent surgery replaced his aortic root and valve with a cryopreserved homograft accompanied by reimplantation of his right coronary artery. Due to disintegration of the left main (LM) ostium from abscess involvement, the LM could not be reimplanted. He received saphenous vein grafts (SVGs) to the left anterior descending (LAD) and circumflex territories, and the LM was oversewn.

Cardiac computed tomography angiography was performed for his new angina, which showed closure of SVG to circumflex, with patent SVG to a large diagonal and severe stenosis of the LAD. The LM stump was approximately 5 mm from the aorta (Figure 1). Catheterization further elucidated a subtotaled diagonal lesion just proximal to the SVG insertion, through which the left coronary system was partially filled, in addition to right-to-left collaterals from the right coronary artery to the circumflex and left-to-left collaterals from the grafted diagonal to the LAD (Figure 2, Videos 1 and 2). After heart team discussion with patient and family involvement, due to the high risk of fourth sternotomy following prior aortic homograft, we elected to attempt PCI.

**Figure 1:**
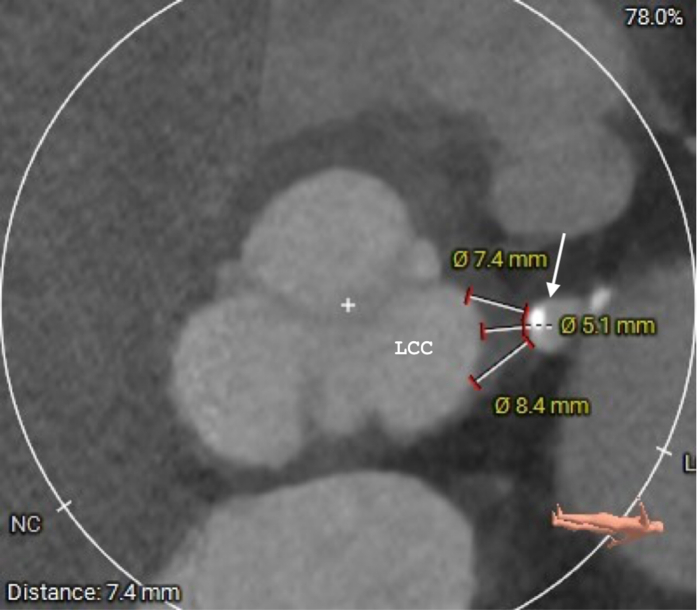
Computed Tomography Showing Distances From Left Main Stump (Arrow) to Aortic Root LCC = left coronary cusp; NC = noncoronary.

**Figure 2:**
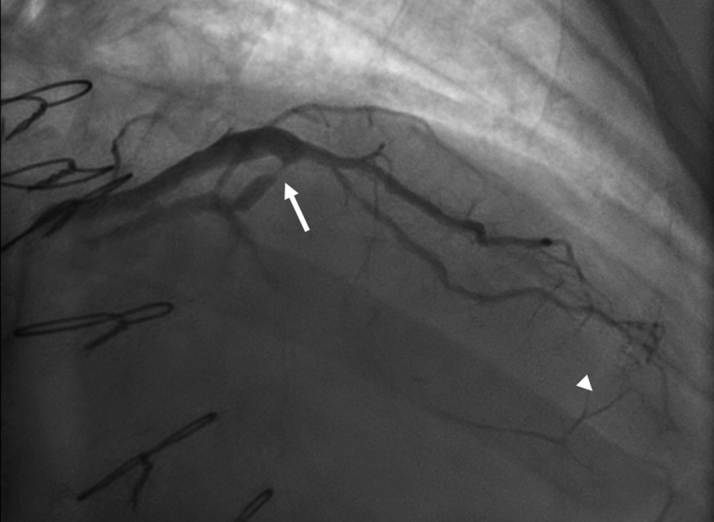
Angiography of Saphenous Vein Graft to Diagonal Showing Severe Stenosis Proximal to Graft Insertion (Arrow) and Left-to-Left Collateralization of Distal Left Anterior Descending (Arrowhead)

Access to the LM was retrograde via the SVG to diagonal, with balloon dilation of the proximal diagonal lesion to prevent intraprocedural ischemia. After penetrating into periaortic adhesions, an Astato 20 (Asahi Intecc) was energized by a unipolar electrosurgery pencil, and the wire advanced until the aortic lumen was reached. Guidewire snaring, externalization, and intravascular ultrasound-guided neo-ostial stenting were performed using standard CTO PCI techniques (Figure 3, Figure 4, Figure 5, Figure 6, Video 3, Video 4, Video 5, Video 6). A 4.5 × 16-mm Megatron drug-eluting stent (Boston Scientific) was placed from the aorta into the proximal LAD given the short landing zone in the LM, and the proximal stent edge was flared in the aorta using an Ostial Flash balloon (Verge Medical). Megatron was chosen due to increased radial strength and was flared to allow for better anchoring and ability for future engagement. Following placement and postdilation with a 4.5-mm noncompliant balloon, IVUS showed focal underexpansion immediately distal to the aortic wall (Figure 5, Video 6); however, owing to concerns about homograft friability and minimal stent area >9 mm^2^, more aggressive postdilation attempts were not made. Final angiography showed normal flow throughout the left coronary system (Videos 4 and 5), including the mid LAD revealed to be collateralized due to underfilling rather than a separate lesion.

**Figure 3:**
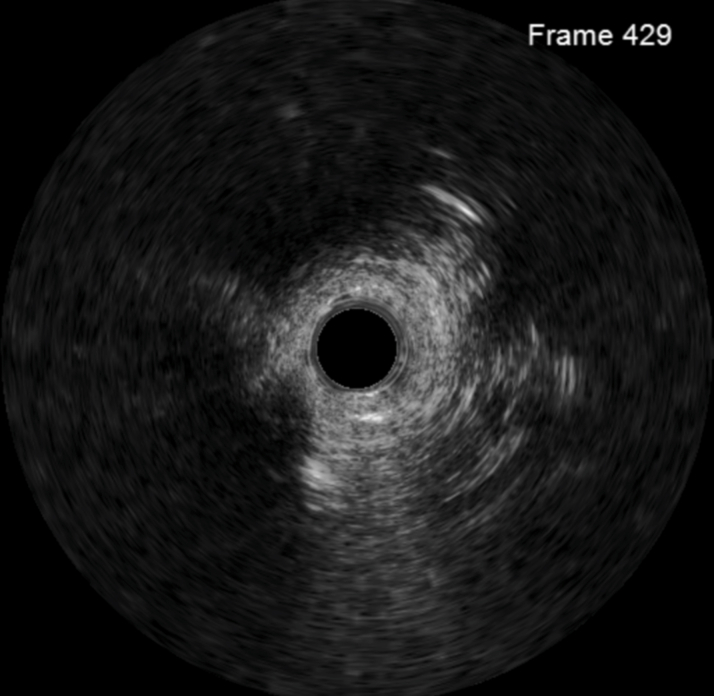
Still-Frame From Intravascular Ultrasound Following 2.0-mm Balloon Dilation Within Periaortic Adhesions Showing Complete Lack of Vascular Structure

**Figure 4:**
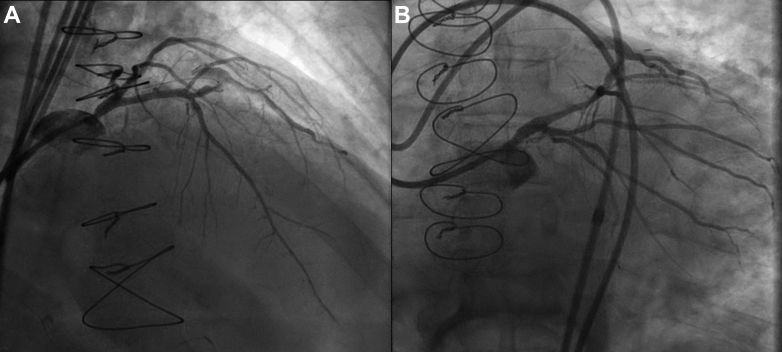
Final Angiography Following Stent Placement (A) Right anterior oblique cranial view; (B) left anterior oblique caudal view.

**Figure 5:**
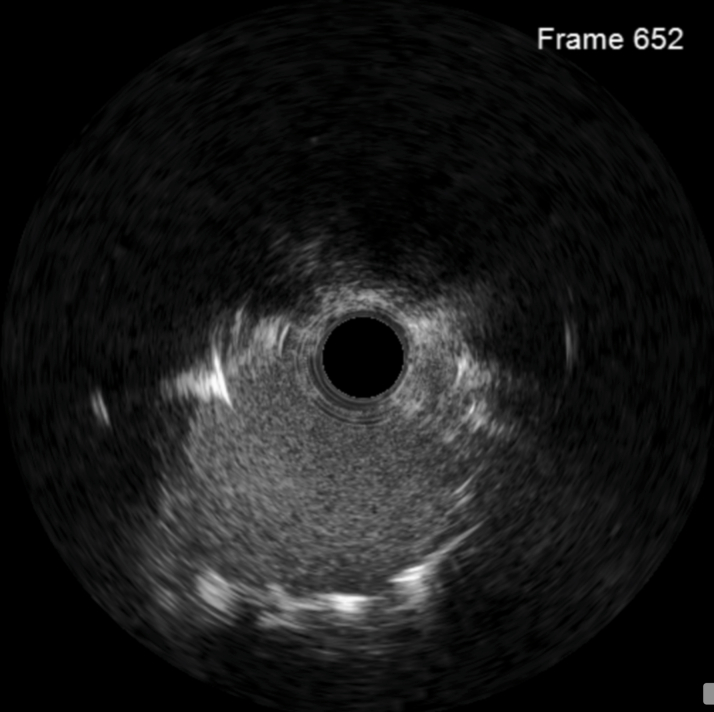
Final Intravascular Ultrasound Following Stent Placement at Location of Minimal Stent Area Just Outside Aorta, Which Was 9.1 mm^2^

**Figure 6:**
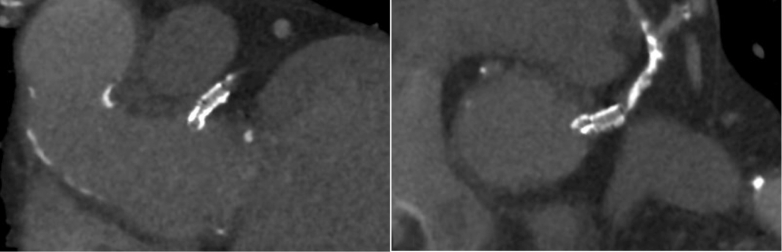
Postprocedural Computed Tomography Showing Orthogonal Views of Stent

Although electrocautery-assisted re-entry has been described in CTO PCI,^1^ our case demonstrates the first use of electrocautery-assisted reentry to traverse a nonvascular space to facilitate creating a neovascular conduit between an aortic root homograft and a surgically ligated LM coronary artery.

## Funding Support and Author Disclosures

Dr Frizzell has received consulting fees/honoraria from Asahi Intecc, Boston Scientific, and Shockwave Medical (Johnson & Johnson). Dr El Hangouche has reported that she has no relationships relevant to the contents of this paper to disclose.

## Appendix

Video 1 Angiography of Saphenous Vein Graft to Diagonal; Note Subtotal Occlusion Proximal to Graft Insertion and Collateralization of Left Anterior Descending

Video 2 Angiography of Reimplanted Right Coronary Artery Showing Right-to-Left Collateralization of the Circumflex

Video 3 Intravascular Ultrasound After 2.0-mm Balloon Dilation, With Pullback From Proximal Left Anterior Descending to Aorta Note disappearance of vascular structures between left main and aorta.

Video 4 Final Angiography Following Stent Placement in Right Anterior Oblique Cranial View Note lack of lesion in the mid left anterior descending with normal antegrade flow.

Video 5 Final Angiography Following Stent Placement in Left Anterior Oblique Caudal View

Video 6 Final Intravascular Ultrasound Following Stent Placement With Pullback From Proximal Left Anterior Descending Into Aorta Guide catheter placed at neo-ostium, although in last few frames, the stent protruding into the aorta is seen.
